# Random survival forests for the analysis of recurrent events for right-censored data, with or without a terminal event

**DOI:** 10.1186/s12874-025-02678-z

**Published:** 2025-11-20

**Authors:** Juliette Murris, Olivier Bouaziz, Michal Jakubczak, Sandrine Katsahian, Audrey Lavenu

**Affiliations:** 1 HeKA, Inria, Inserm, Centre de recherche des Cordeliers, Université Paris Cité, Paris, France; 2 R&D, Pierre Fabre, Boulogne-Billancourt, France; 3 Université Paris Cité, CNRS, MAP5, Paris, F-75006 France; 4 Ardigen S.A, Kraków, Poland; 5 Unité de Recherche Clinique, Hôpital Européen Georges-Pompidou, Assistance Publique – Hôpitaux de Paris (AP-HP), Centre, Paris, France; 6 Centre d’Investigation Clinique 1418 Épidémiologie Clinique, Paris, France; 7 Faculté de Médecine, Université de Rennes, Rennes, France; 8 Institut de Recherche Mathématique de Rennes (IRMAR), Rennes, France; 9 Centre de Investigation Clinique 1414, Inserm, Université de Rennes, Rennes, France

**Keywords:** Random forests, Recurrent events, Survival analyses, Terminal events, High-dimensional data

## Abstract

**Background:**

Random survival forests (RSF) have emerged as valuable tools in medical research. They have shown their utility in modelling complex relationships between predictors and survival outcomes, overcoming linearity or low dimensionality assumptions. Nevertheless, RSF have not been adapted to right-censored data with recurrent events (RE).

**Methods:**

This work introduces RecForest, an extension of RSF and tailored for RE data, leveraging principles from survival analysis and ensemble learning. RecForest adapts the splitting rule to account for RE, with or without a terminal event, by employing the pseudo-score test or the Wald test derived from the marginal Ghosh-Lin model. The ensemble estimate is constructed by aggregating the expected number of events from each tree. Performance metrics involve a concordance index (C-index) tailored for RE analysis, along with an extension of the mean squared error (MSE). A comprehensive evaluation was conducted on both simulated and open-source data. We compared RecForest against the non-parametric mean cumulative function and the Ghosh-Lin model.

**Results:**

Across the simulations and application, RecForest consistently outperforms, exhibiting C-index values ranging from 0.60 to 0.82 and lowest MSE metrics.

**Conclusions:**

As analysing time-to-recurrence data is critical in medical research, the proposed method represents a valuable addition to the analytical toolbox in this domain. The RecForest implementation is publicly available as an R package on CRAN.

**Supplementary Information:**

The online version contains supplementary material available at 10.1186/s12874-025-02678-z.

## Introduction

Recurrent events refer to instances where individuals may experience multiple occurrences of the same event over time. In medical research, patients may face recurrent disease relapses, frequent hospitalizations, or repeated surgeries. While traditional survival analyses focus solely on the first occurrence of an event, specific statistical models have been developed to capture the complexity of recurrence in a survival framework. Intensity models rely on instantaneous hazards at each time point and account for dependence amongst event occurrences captured by time-varying covariates [1, 2]. Besides, marginal models centre on the overall distribution of event times and the cumulative event counts [3, 4]. For a more in-depth exploration of these models concerning recurrent events, comprehensive discussions can be found in works by Amorim (2015) and Ozga (2018) [5, 6].

Time-to-event analyses are systematically challenging due to the presence of censoring, i.e. when the precise timing of an event remains unknown or unobserved. Above methodologies strictly assume the censoring process to be uninformative, hence independent of the underlying event process. Nevertheless, a terminal event may occur in competition, preventing further events of interest from happening. A terminal event is then a specific type of event considered as a termination point for the study period, making the censoring process no longer uninformative. Strategies for handling terminal events include ignoring them, although this approach is acknowledged to be flawed, or accounting for competing risks. Several pertinent statistical models enable to analyse both recurrent events and competing risks [7].

**Fig. 1:**
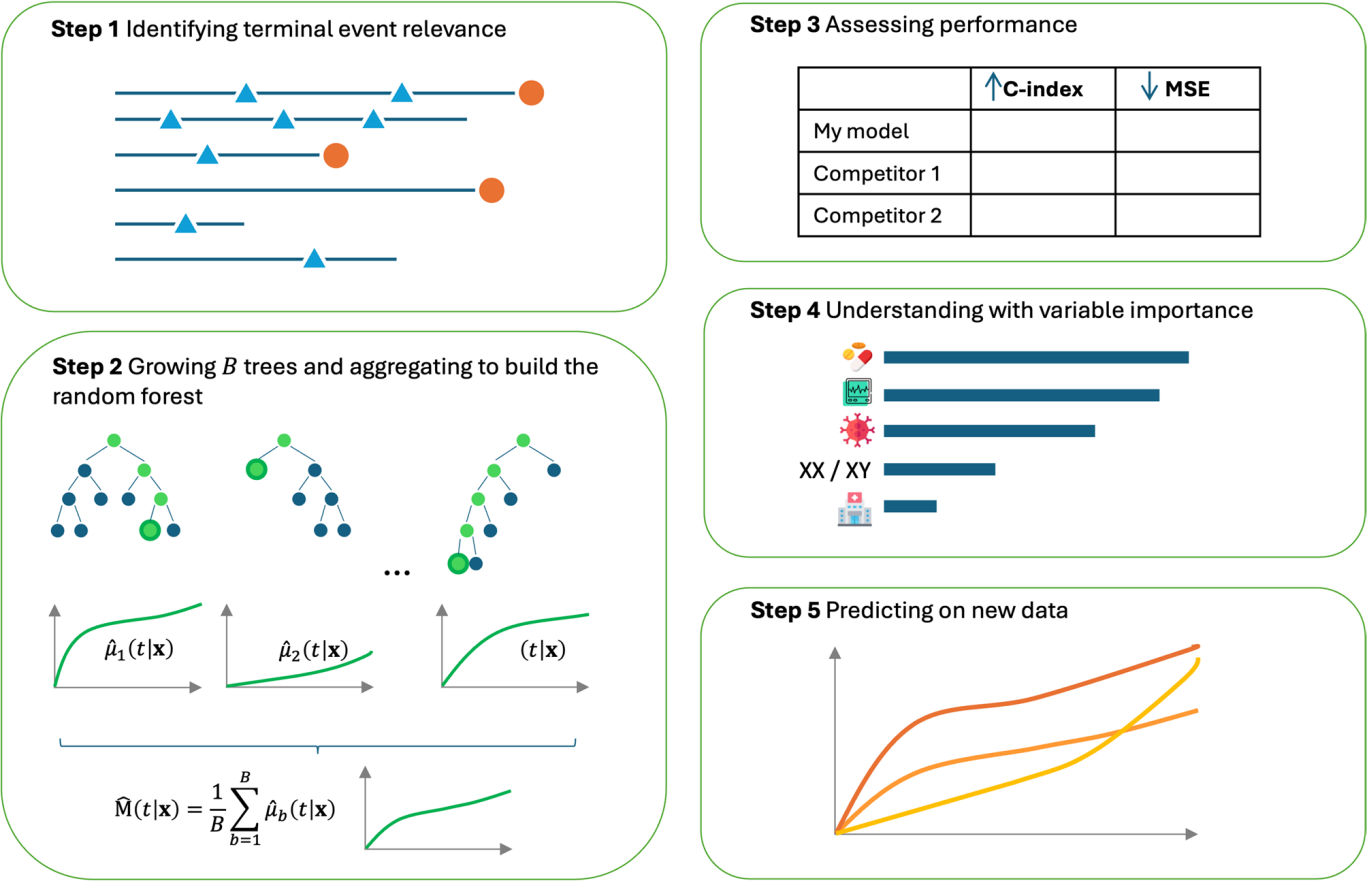
Scheme of the use of RecForest for survival data with recurrent events in presence or absence of a terminal event

Navigating medical data introduces numerous challenges, including high-dimensionality, variable selection, and multicollinearity. To address these, survival time-to-first-event approaches have integrated statistical and machine learning techniques. In practice, various algorithms now have their survival counterparts that are effectively employed to answer medical questions in real-world applications [8]. For instance, penalized regression methods, such as LASSO (Least Absolute Shrinkage and Selection Operator), Ridge, and Elastic-Net, have been tailored for Cox models, facilitating variable selection and regularization [9, 10]. Support-vector machines, renowned for their capacity to handle high-dimensional data and non-linearity, have also been extended to survival endpoints [11]. Likewise, random survival forests (RSF) embody a powerful ensemble learning technique handling interactions [12]. The RSF algorithm has been extended to model several phenomena, such as competing risks, or longitudinal data [13, 14]. However, within the survival framework, no machine learning approach has hitherto been extended to recurrent events [15]. To address these unmet needs and confront the aforementioned challenges, we introduce the first RSF capable of handling recurrent events, with or without a terminal event. Illustrated in Fig.1, our method entails a 5-step approach that *(i)* discerns the relevance of recurrent and terminal events, *(ii)* grows trees to construct a coherent RSF, *(iii)* thoroughly assesses performance, *(iv)* provides relevant variable importance, and *(v)* enables predictions on new data.

In this paper, we consider $\:n$ individuals. Let $\:{T}_{ij}$ be the time of the $\:j$-th recurrent event for subject $\:i=1,...,n$, and $\:{N}_{i}^{\text{*}}\left(t\right)$ denote the true recurrent event counting process over the time interval $\:\left[0,t\right]$, defined by

$$\mathrm{N}_{\mathrm{i}}^{\text{*}}\left(\mathrm{t}\right) = \sum\limits_{j = 1}^{\infty} 1 \left({\mathrm{T}}_{\mathrm{ij}}\leq\mathrm{t}\right).$$.

Let $\:{D}_{i}$ denote the time of a terminal event (e.g., death), with $\:{N}_{i}^{\text{*}}\left(t\right)={N}_{i}^{\text{*}}\left({D}_{i}\right)$ for all $\:t\ge\:{D}_{i}$, and let $\:{C}_{i}$ be the independent right-censoring time. Define $\:{\gamma\:}_{i}=\text{min}\left({D}_{i},{C}_{i}\right)$, and let $\:{{\updelta\:}}_{i}=I\left({D}_{i}<{C}_{i}\right)$ be the terminal event indicator. The observed counting process is then

$$\:{N}_{i}\left(t\right)={N}_{i}^{\text{*}}\left(\text{min}\left(t,{\gamma\:}_{i}\right)\right),$$.

and the observed data consists of $\:\left({N}_{i}\left(t\right),{\gamma\:}_{i},\:{{\updelta\:}}_{i},{X}_{i}\right)$ for $\:i=1,...,n$, where $\:{X}_{i}$ are covariates.

The marginal mean frequency function is defined $\:\mu\:\left(t\right)=\mathbb{E}\left[{N}^{\text{*}}\left(t\right)\right]$, representing the true cumulative number of recurrent events up to time $\:t$. To estimate $\:\mu\:\left(t\right)$, we adopt a unified estimator that accounts for both right-censoring and, when present, terminal events. Let $\:{Y}_{i}\text{}\left(t\right)=1({\gamma\:}_{i}\text{}\ge\:t)$ denote the at-risk indicator for subject $\:i$ at time $\:t$, and let $\:\widehat{S}\left(t\right)$ denote the Kaplan-Meier estimator of the survival function of the terminal event $\:D$. The general form of the estimator is:

$$\:\widehat{{\upmu\:}}\left(t\right)={\int\:}_{0}^{t}{\left(\frac{1}{n}\sum\limits_{i=1}^{n}\frac{{Y}_{i}\left(u\right)}{\widehat{S}\left(u\right)}\right)}^{-1}\left(\frac{1}{n}\sum\limits_{i=1}^{n}\frac{{Y}_{i}\left(u\right)}{\widehat{S}\left(u\right)}\ d{N}_{i}\left(u\right)\right),$$

or more compactly:

$$\:\widehat{{\upmu\:}}\left(t\right)={\int\:}_{0}^{t}\frac{\sum\nolimits_{i=1}^{n}\frac{{Y}_{i}\left(u\right)}{\widehat{S}\left(u\right)}\ d{N}_{i}\left(u\right)}{\sum\nolimits_{i=1}^{n}\frac{{Y}_{i}\left(u\right)}{\widehat{S}\left(u\right)}}.$$

This inverse probability-weighted estimator accounts for the informative censoring induced by a terminal event [16]. In the absence of a terminal event, the survival probability simplifies to $\:\widehat{S}\left(t\right)=1$ for all $\:t$, and the estimator reduces to the Nelson-Aalen estimator for the cumulative mean function [17]:

$$\:\widehat{{\upmu\:}}\left(t\right)={\int\:}_{0}^{t}\frac{\sum\nolimits_{i=1}^{n}{Y}_{i}\left(u\right)\ d{N}_{i}\left(u\right)}{\sum\nolimits_{i=1}^{n}{Y}_{i}\left(u\right)}.$$

From the above considerations arises the evaluation of the provided estimations. Within survival framework, a widely common metric is an extension of the area under the ROC curve known as the concordance index (C-index). The principle of the C-index and its derivatives is to measure the ability of a model to correctly order pairs of survival times [18, 19]. Recent developments have expanded the application of the C-index to the recurrent event framework, incorporating the number of subsequent event occurrences [20]. However, the number of events over time is only comparable if individuals have similar follow-up, which is hardly the case in real-world settings. Therefore, we proposed a generalized C-index by introducing event occurrence rate. Additionally, we employ the mean-square error, recently adapted to account for recurrent events [21].

Based on non-parametric estimators and ensemble method principles, our contribution is to introduce a new ensemble approach, called RecForest, for the analysis of recurrent events in a survival framework, with or without a terminal event. A review of existing methods and related work is provided in Background and related work to contextualize our work. The overall methodology, based on survival decision trees and novel associated evaluation metrics, is detailed in Methods. Simulation study displays an extended simulation study to evaluate the proposed methodology, and Illustrative example provides an illustrative medical application using open-source data.

## Background and related work

### Survival analysis for recurrent events

Survival analysis traditionally focuses on modeling the time until a single event occurs, such as death or disease recurrence. However, many real-world phenomena involve multiple occurrences of the same event over time, necessitating specialized methods for handling recurrent event data. Recurrent event processes arise in various fields, particularly in biomedical research, where individuals may experience repeated hospitalizations, disease relapses, or treatment cycles [22–24].

#### Counting processes and event history

Recurrent event data can be represented using counting processes, where $\:{N}_{i}\left(t\right)$ counts the number of events for subject $\:i$ up to time $\:t$. The event history of a process, also called filtration, captures all past events up to time $\:t$ [22]. This historical perspective is fundamental in influencing model selection between conditional and marginal approaches. Conditional models account for past events through covariates, whereas marginal models assume independence between occurrences.

#### Recurrent event models

Recurrent event models can be broadly categorized based on their underlying assumptions about event dependence and the risk structure. The Andersen-Gill model extends the Cox proportional hazards model by considering the counting process representation of recurrent events [25]. This approach assumes that each event is independent, conditional on observed covariates, making it suitable for scenarios where the dependence between recurrent events is explained entirely by time-dependent covariates. In contrast, the Prentice-Williams-Peterson model introduces a stratified approach where each event is treated as a separate stratum, allowing for varying baseline hazards across different recurrences [26]. This stratification accounts for event order, making the model more suitable when the hazard function is expected to change with each recurrence.

An alternative perspective is provided by frailty models, which introduce an unobserved random effect to account for unmeasured heterogeneity across individuals [27]. These models assume that individuals with a high frailty term are more likely to experience recurrent events, capturing dependencies that are not explained by observed covariates. The frailty component is typically modeled using a gamma distribution, leading to a shared frailty model where the recurrent events of an individual are correlated through the random effect. Such models are particularly useful in medical studies where genetic predisposition or underlying susceptibility factors contribute to repeated event occurrences [5].

Marginal models provide another avenue for analyzing recurrent events by focusing on the mean number of occurrences rather than the hazard function. The Wei, Lin, and Weissfeld model treats each recurrence as an independent process, estimating covariate effects without making assumptions about within-subject correlation [28]. This model is particularly useful when the goal is to assess the overall effect of treatment or exposure on event frequency rather than the timing of each recurrence. The mean cumulative function approach further complements marginal models by estimating the expected cumulative number of events over time, providing an intuitive interpretation of recurrence patterns in a population [17].

The choice of model depends on the research question and data structure (5,7,29). Conditional models like Andersen-Gill are well-suited for time-to-event analyses where covariates explain recurrence dependencies, while marginal models such as Wei-Lin-Weissfeld are preferred when assessing cumulative event counts. Frailty models bridge these approaches by introducing individual-level heterogeneity, making them valuable in scenarios where unobserved factors influence event recurrence. The application of these models spans medical research, reliability analysis, and epidemiology, ensuring that recurrent event processes are appropriately accounted for in survival studies.

#### With terminal events

The presence of a terminal event, such as death, introduces a competing risk scenario where further recurrences become impossible [28, 29]. Traditional recurrent event models assume non-informative censoring, but when a terminal event is present, this assumption is violated, leading to biased estimates if not appropriately addressed. To correct for this, an adjusted mean cumulative function estimator has been proposed, which incorporates survival probabilities [16, 17].

Other approaches for handling terminal events include multi-state models, which explicitly define transitions between recurrent and terminal states, and joint modeling techniques that simultaneously estimate survival and recurrent event processes while accounting for their dependence [30].

### Random survival forest algorithm

Random Survival Forests (RSF) were introduced as an extension of classical random forests to survival data [12]. Unlike traditional parametric models, RSF does not assume a specific form for the survival function, providing greater flexibility across different data distributions.

#### Principle of random survival forests

RSF is based on ensemble learning, where multiple survival trees are built from bootstrap samples of the training data. Each tree is constructed via recursive partitioning using a splitting criterion adapted to censored data. The two main splitting rules commonly used are the log-rank splitting, and the likelihood ratio statistics.

Like classical random forests, RSF leverages bootstrap sampling to generate multiple trees. Observations not included in a given bootstrap sample (out-of-bag or OOB) are used to obtain an internal estimation of the model’s error, eliminating the need for a separate validation set.

#### Terminal node estimator and prediction

In RSF, survival is estimated within each terminal node of the tree. Unlike standard decision trees that assign a class label to a terminal node, RSF constructs a non-parametric Nelson-Aalen estimator for the cumulative hazard function. The survival function for an individual in a terminal node is derived as:

$$\:\widehat{S}\left(t\right)=\text{exp}\left(-\sum\limits_{u\le\:t}\widehat{H}\left(u\right)\right),$$

where $\:\widehat{H}\left(u\right)$ is the cumulative hazard function estimated using the individuals within that node. The final survival prediction for a new observation is obtained by averaging the survival estimates across all trees in the forest. This ensemble approach helps smooth individual tree predictions and improves robustness.

#### Extensions and recent work

Since their introduction, RSF has been extended to address various specific needs. One of the key areas of development has been the adaptation of RSF to competing risks scenarios, where multiple event types exist, and the occurrence of one event precludes the occurrence of another. Ishwaran (2014) proposed an adaptation of RSF for competing risks using Gray’s test, which allows for the estimation of cumulative incidence functions in a non-parametric manner [13]. This extension has significantly improved the applicability of RSF in medical and epidemiological studies where multiple event types, such as different causes of death, must be considered.

Another significant extension of RSF involves dynamic prediction models. Pickett (2021) introduced landmark approaches that enable real-time updates of survival predictions as new covariate information becomes available [31]. This extension is particularly useful in clinical settings where patient characteristics evolve over time. More recently, Devaux (2023) combined dynamic prediction techniques with longitudinal data [14]. This framework enhances RSF’s ability to incorporate time-dependent covariates, making it a powerful tool for personalized medicine and disease progression modeling.

Bayesian approaches have also been integrated into RSF to improve flexibility and incorporate prior information into survival analysis. Chipman (2010) introduced Bayesian Additive Regression Trees (BART), which were later adapted for survival data and incorporated into RSF [32]. These Bayesian RSF models allow for probabilistic inference and uncertainty quantification, making them particularly useful in high-dimensional and sparse data settings. Additionally, Bayesian methods facilitate variable selection and regularization, helping to address overfitting issues in complex survival datasets.

Beyond these advances, RSF has also been extended for high-dimensional variable selection. Ishwaran (2010) introduced RSF-VH (Variable Hunting), which ranks variables based on their minimal depth within survival trees, providing a non-parametric approach to feature selection [33]. Furthermore, Genuer (2010) proposed a structured variable selection process that balances interpretability and predictive accuracy [34]. Another key contribution introduced regularization techniques to refine feature selection and improve the model’s robustness against irrelevant or redundant variables [35].

Finally, ongoing research is focusing on the computational efficiency and scalability of RSF, particularly for large-scale biomedical datasets. Efforts are being made to parallelize RSF algorithms and optimize tree construction to reduce computational overhead, making RSF more accessible for real-time clinical decision-making and large-scale genomic studies.

## Methods

The proposed Algorithm 1 of RecForest is an extension of the RSF introduced by Ishwaran (2008) [12]. The first step consists in drawing bootstrap samples to prevent overfitting and capture inherent variability within the original dataset. Then, survival trees are constructed on each bootstrap sample. Unlike the original RSF, our approach accommodates for subsequent events by integrating statistical considerations tailored for recurrent events analysis. As a last step, the algorithm aggregates the results over the constructed recursive survival trees to obtain a comprehensive estimate.

### Algorithm 1 Overview of recforest algorithm

**Table:** 

(1) Draw $B$ bootstrap samples from the learning data;
(2) Draw a survival tree $b$ extended to recurrent events; At each node, • $mtry$ predictors are randomly selected with $mtry$ $\in$ $\mathbb{N}$, $mtry$ ≤ 𝑝; • A greedy algorithm for optimal threshold research is used to maximize the test statistic; The tree grows until the stopping rule is met based on the minimal number of events 𝑚𝑖𝑛𝑠𝑝𝑙𝑖𝑡 and the minimal number of individuals in terminal nodes *nodesize*; Estimate $\widehat\mu\ _{b}$is computed;
(3) Estimate $\widehat {\text M}$ is computed over the $B$ trees

Next subsections describe in further details how survival trees grow for constructing the random forest. Additionally, we provide adequate metrics for the evaluation. Finally, we expound on the computation of variable importance.

### Growing trees with recurrent events

#### Splitting rules

At each node $\:h\in\:\mathcal{H}$, the ongoing subsample is split into two daughter nodes denoted $\:{h}^{\left(+\right)}$ and $\:{h}^{\left(-\right)}$. The aim of the split is to make the daughter nodes as different as possible with regards to the outcome. The splitting rule requires that each of the $\:mtry$ randomly drawn variable is dichotomized. For continuous variables, random split points, quartiles, and deciles are considered. Let $\:{\mathbf{x}}_{h}=A,B$ be the dichotomized vector of a variable inherited from $\:h$. For each split, we compare the marginal mean functions $\:{\mu\:}_{A}\left(t\right)$ and $\:{\mu\:}_{B}\left(t\right)$ between the two groups $\:A$ and $\:B$. The null hypothesis is their equality.

In absence of a terminal event, we use the two-sample test akin to the log-rank test [36]. The test statistic writes $\:U\left(t\right)={\int\:}_{0}^{t}\frac{{Y}_{A}\left(u\right){Y}_{B}\left(u\right)}{{Y}_{A}\left(u\right)+{Y}_{B}\left(u\right)}\left(\text{d}{\widehat{\mu\:}}_{A}\left(u\right)-\text{d}{\widehat{\mu\:}}_{B}\left(u\right)\right)$, with $\:{Y}_{A}\left(t\right)$ and $\:{Y}_{B}\left(t\right)$ the number at risk in each group at time $\:t$.

In the presence of a terminal event, we follow the marginal model from Ghosh-Lin (GL) within the single variable $\:{\mathbf{x}}_{h}$ [37]. Acknowledging there are no further recurrence after the terminal event, the marginal mean up to $\:t$ associated with $\:{\mathbf{x}}_{h}$ is defined as $\:{\mu\:}_{{\mathbf{x}}_{h}}\left(t\right)=\mathbb{E}\left[{N}^{\text{*}}\left(t\right)|{\mathbf{x}}_{h}\right]={\mu\:}_{0}\left(t\right)\times\:\text{exp}\left(\beta\:{\mathbf{x}}_{h}\right)$ with $\:{\mu\:}_{0}$ left unspecified and $\:\beta\:$ the regression coefficient. To accommodate longitudinal variables, the model follows the marginal approach of Ghosh-Lin and we adopt the marginal rate function $\:{\text{d}\mu\:}_{{\mathbf{x}}_{h}}\left(t\right)={\text{d}\mu\:}_{0}\left(t\right)\times\:\text{e}\text{x}\text{p}\left(\beta\:{\mathbf{x}}_{h}\left(t\right)\right)$, where $\:{\mathbf{x}}_{h}\left(t\right)$ may vary over time. The Wald test statistic is then extracted from $\:{\mu\:}_{{\mathbf{x}}_{h}}$ and $\:{\text{d}\mu\:}_{{\mathbf{x}}_{h}}$ to test the null hypothesis of $\:\beta\:=0$. The variable selected for node $\:h$ is the one that maximizes the adequate test statistic to generate $\:{h}^{\left(+\right)}$ and $\:{h}^{\left(-\right)}$, based on the presence of a terminal event and/or longitudinal variables.

#### Terminal node estimator

Let $\:{\mathcal{B}}_{b}$ be a bootstrap sample drawn from original data on which a tree $\:b$ is grown, and let $\:\mathbf{x}$ be a $\:p$-dimensional covariate vector dropped down the tree. Individuals with similar covariate trajectories are grouped into the same terminal node, denoted $\:{\mathcal{T}}_{b}\left(\mathbf{x}\right)$, which contains the subset of individuals assigned to the same terminal node as $\:\mathbf{x}$ in tree $\:b$. For each terminal node, we define a node-specific estimator $\:{\widehat{\mu\:}}_{{\mathcal{T}}_{b}}\left(t|\mathbf{x}\right)$ as the estimated number of recurrent events up to time $\:t$, based on the individuals in $\:{\mathcal{T}}_{b}\left(\mathbf{x}\right)$. This estimator is given by:

$$\:{\widehat{\mu\:}}_{{\mathcal{T}}_{b}}\left(t|\mathbf{x}\right)={\int\:}_{0}^{t}\frac{\sum\nolimits_{i\in\:{\mathcal{T}}_{b}\left(\mathbf{x}\right)}\frac{{Y}_{i}\left(u\right)}{{\widehat{S}}_{{\mathcal{T}}_{b}\left(\mathbf{x}\right)}\left(u\right)}\ d{N}_{i}\left(u\right)}{\sum\nolimits_{i\in\:{\mathcal{T}}_{b}\left(\mathbf{x}\right)}\frac{{Y}_{i}\left(u\right)}{{\widehat{S}}_{{\mathcal{T}}_{b}\left(\mathbf{x}\right)}\left(u\right)}},$$

Where $\:{Y}_{i}\left(t\right)$ is the at-risk indicator for subject $\:i\in\:{\mathcal{T}}_{b}\left(\mathbf{x}\right)$, and $\:{\widehat{S}}_{{\mathcal{T}}_{b}\left(\mathbf{x}\right)}$ is the Kaplan-Meier estimator of the survival function, estimated using the subset $\:{\mathcal{T}}_{b}\left(\mathbf{x}\right)$.

In the absence of a terminal event, we have $\:{\widehat{S}}_{{\mathcal{T}}_{b}\left(\mathbf{x}\right)}\left(t\right)=1$ for all $\:t$, and the estimator simplifies to the Nelson-Aalen form:

$$\:{\widehat{\mu\:}}_{{\mathcal{T}}_{b}}\left(t|\mathbf{x}\right)={\int\:}_{0}^{t}\frac{\sum\nolimits_{i\in\:{\mathcal{T}}_{b}\left(\mathbf{x}\right)}{Y}_{i}\left(u\right)\ d{N}_{i}\left(u\right)}{\sum\nolimits_{i\in\:{\mathcal{T}}_{b}\left(\mathbf{x}\right)}{Y}_{i}\left(u\right)}.$$

This terminal-node specific estimator captures the recurrent event dynamics of individuals with similar covariate patterns, and forms the building block for constructing ensemble estimators.

#### Pruning trees

A pruning strategy is essential to help find a trade-off to prevent overfitting and improve generalization performance of trees, within a reasonable computational time. Aligned with Devaux (2023), we suggest two stopping rules for each terminal node: *(i)* a minimal number of events called $\:minsplit$, and *(ii)* a minimal number of individuals called $\:nodesize$ [14]. The validation of either stopping rule designates the current node $\:h$ as terminal.

#### Handling missing data

To tackle eventual missing data, we include an adaptive-tree imputation which addresses missing data during the tree-growing stage by selectively drawing from available, non-missing, in-bag data [12, 38]. At each node $\:{h}_{b}$ from tree $\:b$, the method entails imputing random non-missing information specifically from the selected variables. The imputed data is then utilized for making splits within the node $\:{h}_{b}$. Imputed values are reset to missing as the tree progresses to subsequent nodes.

### From trees to random forests

#### Ensemble estimates

Once all $\:B$ trees are grown from the $\:{\mathcal{B}}_{b=1,...,B}$independent bootstrap samples, we aggregate the predictions from each tree to form the final ensemble estimate. For a given covariate vector $\:\mathbf{x}$, let $\:{\widehat{\mu\:}}_{b}$ denote the marginal mean function estimated from terminal nodes $\:{\mathcal{T}}_{b}\left(\mathbf{x}\right)$. The ensemble estimate $\:\widehat{\text{M}}$ is the aggregation of all $\:B$ tree-specific estimates:

$$\widehat{\text{M}}\left(\left.t\right|\mathbf{x}\right)=\frac1B\sum\limits_{b=1}^B{\widehat\mu}_b\left(\left.t\right|\mathbf{x}\right)$$

This ensemble prediction leverages the variability captured by bootstrap sampling and the recursive partitioning structure of each tree to smooth out individual tree estimators and improve predictive stability. Of note, in case of no terminal event, the ensemble estimator provides a nonparametric estimate of the cumulative expected number of recurrent events.

#### OOB ensemble estimates

By bootstrap sampling theory, each tree in the forest is trained on a subset of the data, leaving out roughly 37% of the original observations, called the out-of-bag (OOB) sample. These OOB samples are used to construct OOB ensemble estimates, which serve as internal validation predictions without the need for a separate test set.

Let $\:{\mathcal{B}}_{b}\text{}\:$denote the bootstrap sample used to build tree $\:b\text{}$, and define $\:{\mathcal{O}}_{i}=\{b:i\notin\:{\mathcal{B}}_{b}\}$ as the set of trees for which individual $\:i$ is out-of-bag. The OOB ensemble estimator for the set of individuals with covariate vector $\:\mathbf{x}$ is given by:

$$\widehat{\text{M}}^{OOB}\left(\left.t\right|\mathbf{x}\right)=\frac1{\left|O_i\right|}\sum\limits_{b\in O_i}{\widehat\mu}_b\left(\left.t\right|\mathbf{x}\right),$$

where $\:{\widehat{\mu\:}}_{b}\left(t|\mathbf{x}\right)$ is the marginal mean function estimated from terminal nodes $\:{\mathcal{T}}_{b}\left(\mathbf{x}\right)$, defined as in the prior section. The OOB ensemble prediction thus averages only the tree-specific predictions from trees where the subject was not included in training.

These OOB predictions are particularly useful for assessing model performance, tuning hyperparameters, or computing variable importance, especially in settings where external validation sets are unavailable or expensive to reserve.

### Performance

Performance metrics below indicate the ability of the model to predict well from training data to unseen data. In our case, unseen data are either from the OOB sample or external validation data.

#### Assessing performance with relevant metrics

**Fig. 2:**
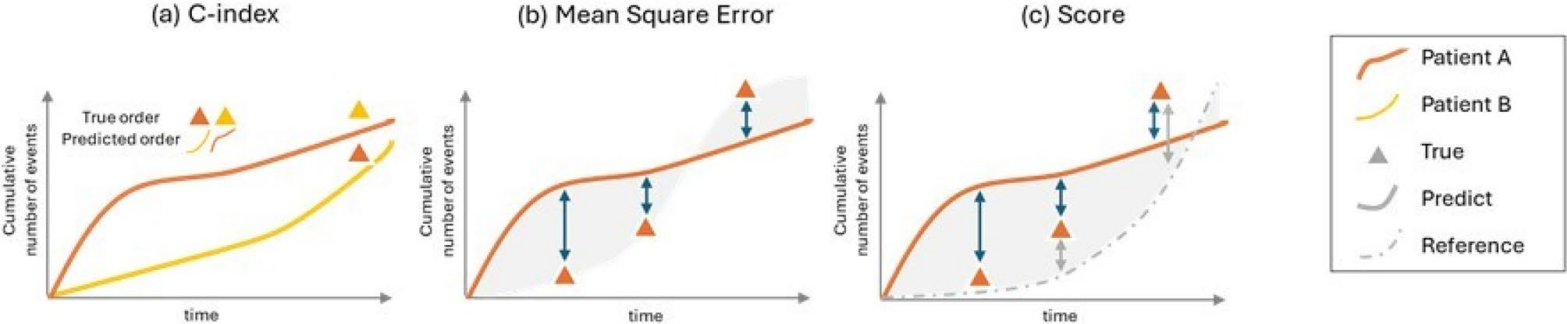
Illustration of the performance metrics with true and predicted cumulative number of events over time

For the assessment of performance, we introduce an extended version of the C-index and employ the mean-square error (MSE), a derived score, and their integrated versions (Fig. 2).

##### Concordance index

Kim (2018) adapted the C-index to recurrent events and considered the number of events over time across individuals [20]. This metric hence suffers from the potential bias in case of substantial variability in the follow-up times [15]. Individuals with longer follow-up times or a higher number of events might indeed disproportionately influence the C-index calculation. To address this issue, we suggest using occurrence rates by computing event rates per unit time.

The proposed C-index is defined as the proportion of all concordant pairs of individuals where predicted occurrence rates are correctly ordered with respect to observed occurrence rates (as shown in Fig. 2(a)). As occurrence rates can be calculated for all individuals, including censored ones, the proposed C-index is not partial and considers all individuals in the computation. For each individual $\:i=1,\dots\:,n$, we define their observed rate as the total number of observed events divided by their observed time, $\:{r}_{i}={N}_{i}\left({{\upgamma\:}}_{i}\right)/{{\upgamma\:}}_{i}$ and $\:{\widehat{r}}_{i}=\widehat{\text{M}}\left({\gamma\:}_{i}|{\mathbf{x}}_{i}\right)/{{\upgamma\:}}_{i}$, respectively, where $\:{{\upgamma\:}}_{i}=\text{min}\left({D}_{i},{C}_{i}\right)$ is the observed time (i.e., minimum of terminal event or censoring), and $\:\widehat{{\text{M}}}\left({\gamma\:}_{i}|{\mathbf{x}}_{i}\right)$ is the predicted cumulative number of events. In this work, the C-index is calculated as the proportion of all comparable pairs $\:(i,j)$ where the ordering of $\:{\widehat{r}}_{j}$ and $\:{\widehat{r}}_{i}$​ matches that of $\:{r}_{i}$​ and $\:{r}_{j}$​. The C-index then writes

$$\widehat{\mathbb{C}}\left(\widehat{\mathrm M}\right)=\frac{\sum_{i=1}^n\sum_{j=1}^nI\left(r_i>r_j\right)\times I\left({\widehat r}_i>{\widehat r}_j\right)}{\sum_{i=1}^n\sum_{j=1}^nI\left(r_i>r_j\right)}.$$

Like other C-indices, the value of the above C-index falls within the range of 0 to 1, where 1 indicates perfect concordance, and values close to 0.5 suggest randomness in the model.

#####  Mean-squared error and derived score

No MSE measure has been adapted to recurrent events framework until very lately. Bouaziz (2023) filled this gap and suggested a generalization of the Brier score from Graf (2000) [21, 39]. For our problematic, for each tree 

$\:b$ and $\:{\widehat{\mu\:}}_{b}$ the marginal mean function estimated, we define

$$\:{\widehat{MSE}}_{b}\left(t,{\widehat{\mu\:}}_{b}\right)=\frac{1}{n}\sum\nolimits_{i=1}^{n}{\left({\int\:}_{0}^{t}\frac{\text{d}{N}_{i}\left(u\right)}{{\widehat{G}}_{c}\left(u|\mathbf{x}\right)}-{\widehat{\mu\:}}_{b}\left(t|\mathbf{x}\right)\right)}^{2},$$.

where $\:{\widehat{G}}_{c}\left(u|\mathbf{x}\right)=1-\widehat{G}\left(u-|\mathbf{x}\right)$ is an estimator of $\:{G}_{c}\left(u|\mathbf{x}\right)=1-G\left(u-|\mathbf{x}\right)$ the conditional cumulative distribution function of the censoring variable $\:C$ given $\:\mathbf{x}$. If there is no terminal event, the censoring distribution $\:G$ is estimated using the empirical cumulative distribution function of the censoring times, since all censoring times are fully observed. In the presence of a terminal event, however, $\:C$ becomes incompletely observed due to the competing risk of the terminal event $\:D$. In this case, $\:G$ is estimated using the Kaplan-Meier estimator, treating terminal events as censoring events. Moreover, since assume $\:C$ and $\:\mathbf{x}$ to be independent, the conditional survival function $\:G\left(t|\mathbf{x}\right)$ simplifies to the marginal $\:G\left(t\right)$, and we denote its estimate by $\:\widehat{G}\left(t\right)$. As suggested in Fig. 2(b), the general prediction criterion denoted $\:\widehat{MSE}$ over our random forest hence writes

$$\:\widehat{MSE}\left(t,\widehat{\text{M}}\right)=\frac1B\sum_{b=1}^B{\widehat{MSE}}_b{(t,\widehat{\mu\:}}_b).$$

However, as pointed out in Bouaziz (2023), two different models may lead to similar MSE values over time due to the inseparability term (noise inherent in the process), thus underlining the difficulty in assessing which model is better [21]. Therefore, a score is introduced to represent the prediction gain compared to a reference estimator and we define for each tree $\:b$:

$$\:{Score}_{b}\left(t,{\widehat{\mu\:}}_{b},{\widehat{\mu\:}}_{b,0}\right)={\widehat{MSE}}_{b}\left(t,{\widehat{\mu\:}}_{b,0}\right)-{\widehat{MSE}}_{b}\left(t,{\widehat{\mu\:}}_{b}\right),$$

where $\:{\widehat{\mu\:}}_{b}$ is the evaluated estimator and $\:{\widehat{\mu\:}}_{b,0}$ the reference estimator over the $\:b$ samples. In our case, the reference estimator is the tree-specific non-parametric either the Nelson-Aalen or the Ghosh-Lin estimator described above. The ensemble score illustrated in Fig. 2(c) writes a higher score is associated with a better performance.

$$\:Score\left(t,\widehat{\text{M}}\right)=\frac1B\sum_{b=1}^B{Score}_b\left(t,{\widehat{\mu\:}}_b,{\widehat{\mu\:}}_{b,0}\right)$$

#####  Integrated counterparts

Above MSE and derived score are time-dependent metrics. While they provide valuable insight of the performance for each time 

$\:t$, there is a need for the estimation of the expectation of single-time MSE and derived score over time (shaded areas in Fig. 2). As demonstrated in Bouaziz (2023), above MSE reduces to the Brier score when individuals experience one event at most [21]. In the spirit of the integrated version of the Brier score between two time points $\:{\tau\:}_{1}$ and $\:{\tau\:}_{2}$, we integrate the MSE and the score:

$$\left\{\begin{array}{l}\widehat{\mathrm{IMSE}}\left({\tau\:}_1,{\tau\:}_2,\widehat{\mathrm M}\right)=\frac1{{\tau\:}_2-{\tau\:}_1}\int_{{\tau\:}_1}^{{\tau\:}_2}\widehat{\mathrm{MSE}}\left(t,\widehat{\mathrm M}\right)dt,\\\mathrm{IScore}\left({\tau\:}_1,{\tau\:}_2,\widehat{\mathrm M}\right)=\frac1{{\tau\:}_2-{\tau\:}_1}\int_{{\tau\:}_1}^{{\tau\:}_2}\mathrm{Score}\left(t,\widehat{\mathrm M}\right)dt,\end{array}\right.$$

with typically $\:{\tau\:}_{1}=0$ and $\:{\tau\:}_{2}$ the maximum event time on the original sample.

#### OOB errors

OOB errors are used for tuning hyperparameters and evaluating predictive performances and are computed on OOB samples. They are also particularly useful in the absence of external validation data or when dealing with low-dimensional original samples, where allocating a portion for validation is hardly affordable. OOB predictions are calculated by average predictions from OOB trees, and the error rate is complementary to 1. In this work, we consider the IMSE to assess the OOB error:

$$\:{OOB\:error=\widehat{IMSE}}^{OOB}\left(t,{\widehat{\text{M}}}^{OOB}\right)$$

In this way, models exhibiting lower OOB errors are consistently favored. Of note, computing OOB errors is not recommended when the number of trees is low as each one of them may underfit.

### Variable importance

The importance of a variable (VImp) is evaluated by permutation, corresponding to the impact of random perturbations in the sample on the OOB error [40]. To quantify the VImp of a covariate, a performance metric, as previously defined, is calculated following the permutation of values associated with this covariate. The VImp is determined as the difference between the original and permuted performance metrics. For covariate $\:j$ and considering $\:K$ permutations, $\:VImp\left(j\right)$ writes

$$\:VImp\left(j\right)=\frac{1}{K}\sum\:_{k=1}^{K}\left(\widehat{\theta\:}-{\widehat{\theta\:}}_{k}^{j}\right)$$.

With $\:\widehat{\theta\:}=\left\{-\widehat{IMSE},\widehat{\mathbb{C}}\right\}$ the original performance metric and $\:{\widehat{\theta\:}}^{j}$ the permutated performance metric. High relative values of VImp indicate a loss of performance and lower/null values are interpreted as no importance for such covariates.

## Simulation study

We propose the following simulation settings to illustrate the use of RecForest, inspired by [13, 21]. Simulation scenarios will cover multiple cases with associated covariates, with or without a terminal event, low- and high-dimensional data, and with or without missing data. 250 learning sets and one external validation set were generated for each scenario with $\:n=250$ individuals and $\:p$ covariates. Next subsections further detail simulation parameters for each case.

For each scenario, we grow 100 trees for RecForest. To ensure comparability across scenarios and reduce computational burden over 250 replications, we fixed $\:nodesize=10$ and $\:minsplit=5$, based on preliminary tuning that balanced tree depth with computational efficiency. We evaluated RecForest using three different values of $\:mtry\in\:\left\{1,\sqrt{p},\text{log}\left(p\right)\right\}$ to assess performance sensitivity to feature subsampling.

We compared RecForest with non-parametric estimators, as well as a semi-parametric GL model where possible. Performances were measured on the external validation set using the C-index, MSE, the score and their integrated versions.

### Simulation scheme

#### With and without a terminal event

For $\:i=1,\dots\:,n$, $\:{p}_{0}$-dimensional covariate vector, $\:{X}_{i}=\left({X}_{i,1},\dots\:,{X}_{i,{p}_{0}}\right)$ is generated, where the first half of the components ${X_{i,1:}}_{\left[\frac{P_o}2\right]\sim B\left(0.5\right)}$ are Bernoulli variables. The remaining covariates $\:{X}_{i,\lfloor\frac{{p}_{0}}{2}\rfloor+1:{p}_{0}}$ are generated from a multivariate normal distribution with $\:{X}_{i,\lfloor\frac{{p}_{0}}{2}\rfloor+1:{p}_{0}}\sim\:{\mathcal{N}}_{{p}_{0}-\lfloor\frac{{p}_{0}}{2}\rfloor}\left(2,\:0.5\cdot\:{\Sigma\:}\left({\uprho\:}\right)\right)$, where $\:{\Sigma\:}\left({\uprho\:}\right)$ is a covariance matrix with an autoregressive structure such that $\:{{\Sigma\:}}_{jk}={{\uprho\:}}^{\left|j-k\right|}$, and $\:{\uprho\:}\in\:\:\left[\text{0,1}\right]$ controls the level of correlation among covariates. Such design allows to evaluate robustness under varying levels of multicollinearity among (continuous) covariates.

Recurrent events are simulated from a non-homogenous Poisson process with intensity $\:\lambda\:\left(t|{X}_{i}\right)={\lambda\:}_{0}\left(t\right)\text{exp}\left({\beta\:}^{\text{T}}{X}_{i}\right)$, where the baseline hazard is Weibull with $\:{\lambda\:}_{0}\left(t\right)=\frac{\alpha\:}{\gamma\:}{\left(\frac{t}{\gamma\:}\right)}^{\alpha\:-1}$, $\:\alpha\:=2$ (shape parameter), and $\:\gamma\:=0.39$ (scale parameter). The vector of coefficients is $\:\beta\:={\left({\beta\:}_{1},\dots\:,{\beta\:}_{{p}_{0}}\right)}^{\text{T}}$ and $\:{\beta\:}_{1}=\text{log}\left(5\right)$, $\:{\beta\:}_{2:4}=\text{log}\left(1.3\right)$, $\:{\beta\:}_{5:{p}_{0}}=\text{log}\left(0.7\right)$. The true expected number of events is given by $\:{\mu\:}^{\star\:}\left(t|{X}_{i}\right)={\int\:}_{0}^{t}\lambda\:\left(u|{X}_{i}\right)\text{d}u={\left(\frac{t}{\gamma\:}\right)}^{\alpha\:}\text{exp}\left({\beta\:}^{\text{T}}{X}_{i}\right)$. The censoring process is then simulated from a uniform distribution $\:\mathcal{U}\left(\text{0,3}\right)$. With a terminal event, the recurrent event process and covariates are simulated in the same way as above.

The censoring process is simulated based on a uniform distribution $\:\mathcal{U}\left(\text{0,8}\right)$, and the terminal event is simulated using a Cox model with the same covariates and coefficients $\:\beta\:$ with shape parameter 8 and scale parameter 1.8. We set $\:{p}_{0}=10$.

#### Low- and high-dimensional scenarios

To define low- and high-dimensional scenarios, we introduce $\:q$ independent noise covariates randomly drawn from a standard normal distribution and add them to simulated datasets. We set $\:q=10$ for low-dimensional scenarios, and $\:q=290$ in high-dimensional scenarios. The total number of covariates for each scenario is $\:p={p}_{0}+q$.

##### Complete

When we analyze scenarios that involve all $\:p$ generated covariates, we refer to these as ‘complete’ datasets analyses. Besides, we set $\:\rho\:\in\:\{0,\:0.2,\:0.5\}$.

##### Missing

To simulate real-world conditions where datasets may have missing values, we intentionally introduced missing data. Specifically, we randomly set 5% of the covariate$\:\:{X}_{1}$ to NA across all individuals. This was done in a completely random manner, ensuring that the missing data does not follow any pattern and is not dependent on any other variables or the values of $\:{X}_{1}$ itself. The missing data mechanism is completely at random. We set $\:\rho\:\:=\:0$. We refer to such scenarios as ‘Missing’.

##### Random

We created scenarios where the covariates are generated independently of the recurrent events to simulate a situation where no underlying factors influence the counting process. In such cases, all $\:\beta\:$ were set to 0. We set $\:\rho\:\:=\:0$. We refer to these scenarios as ‘Random’.

Table 1 below summarizes investigated scenarios.

**Table 1:** Summary of investigated scenari os

	Without a terminal event		With a terminal event	
	Low dimensional n=250	High dimensional n=250	Low dimensional p=20	High dimensional p=300
Complete,$\:\rho\:\in\:\{0,\:0.2,\:0.5\}$	x	x	x	x
Missing	x	x	x	x
Random	x	x	x	x

*ρ *controls the level of correlation among covariates within the covariance matrix$\Sigma(\rho)$

### Results

For each simulation scenario, we generated 250 independent training datasets and one external validation set. Models were trained independently on each dataset (using fixed hyperparameter values for RecForest) and predictions were evaluated on the common external validation set. Performance metrics such as the C-index and integrated score were aggregated across the 250 replicates. The non-parametric estimator uses no covariates, regardless of the dimensionality by construction. For the GL model, no variable selection was performed, meaning all $\:p$ covariates were included in the model. This limits the analysis for the GL model to low-dimensional scenarios only.

#### Without a terminal event

On average, 62% of the individuals experienced at least one recurrent event, 46% had at least two recurrent events, 26% had at least five recurrent events, and circa four recurrent events per individual.

Overall, performances based on C-index values are greater in scenarios with neither missing data, nor high-dimensionality, both in average and in variability (Table 2). As expected, scenarios with random inputs lead to randomness with C-index values neighboring 0.50 for each model. The non-parametric estimator provides an average C-index of 0.55. The GL model seems to suffer from not being well-specified, with average C-index values ranging from 0.49 to 0.55 where assessable. RecForest consistently outperforms the Ghosh–Lin model across all scenarios. In the low-dimensional setting ($\:p=20$), RecForest achieves a higher C-index, particularly in the uncorrelated case ($\:\rho\:=0$) where it reaches up to 0.71 (random scenarios are not deemed for comparing performance). Although the C-index slightly decreases as correlation increases ($\:\rho\:=0.2,\:0.5$), RecForest maintains values around 0.57–0.60, showing robustness to multicollinearity. In the high-dimensional setting ($\:p=300$), RecForest maintains strong discrimination (C-index=0.68–0.70) across all levels of correlation. These results confirm that RecForest is effective in ranking individuals by risk, even under complex, correlated, or high-dimensional covariate structures. Besides, it is not impacted by the introduction of massive noisy data, as C-index values remain similar across low- and -high-dimensional scenarios.

Table 3 outlines performances in terms of integrated scores. As checked with C-indices, there is no expectations in the interpretation of the random scenarios, hence there are not displayed. The non-parametric estimator is the reference model in the computation of the score. RecForest demonstrates clear advantages over the Ghosh–Lin model, particularly in the presence of multicollinearity. In the low-dimensional setting, Ghosh–Lin’s scores deteriorate with increasing correlation, turning negative at $\:\rho\:=0.2$ and $\:\rho\:=0.5$, indicating poor calibration. In contrast, RecForest remains stable and well-calibrated, with scores improving slightly as correlation increases, especially for lower mtry values. In the high-dimensional setting, RecForest continues to deliver high scores even as ρρ increases, showing minimal degradation and outperforming its performance in the independent case ($\:\rho\:=0$). These findings confirm RecForest’s robustness to correlated predictors and its ability to produce accurate and well-calibrated predictions in both low- and high-dimensional scenarios. Notably, RecForest achieved better calibration under missing data than under complete data, as reflected in higher integrated score values. Our intuition is the regularizing effect of imputation may stabilize predictions in the presence of noisy or weakly informative covariates.

**Table 2:** Means and standard deviations of the C-index without a terminal event

Scenario \ Method	Np	GL		RecForest$\:mtry=1$		RecForest$\:mtry=\sqrt{p}$		RecForest$\:mtry=\text{log}\left(p\right)$
Low dimensional		$\:\left\{n=250,p=20\right\}$						
Complete,$\:\rho\:=0$	0.55 (0.04)	0.55 (0.12)		0.68 (0.08)		0.71 (0.04)		0.70 (0.05)
Complete,$\:\rho\:=0.2$		0.54 (0.12)		0.59 (0.21)		0.60 (0.15)		0.59 (0.17)
Complete,$\:\rho\:=0.5$		0.54 (0.15)		0.58 (0.24)		0.57 (0.10)		0.58 (0.16)
Missing		0.52 (0.10)		0.65 (0.14)		0.69 (0.15)		0.67 (0.15)
Random		0.45 (0.09)		0.52 (0.11)		0.53 (0.16)		0.53 (0.12)
High dimensional		$\:\left\{n=250,p=300\right\}$						
Complete,$\:\rho\:=0$		/		0.67 (0.21)		0.70 (0.11)		0.70 (0.17)
Complete,$\:\rho\:=0.2$		/	0.65 (0.25)		0.67 (0.13)		0.70 (0.17)	
Complete,$\:\rho\:=0.5$		/	0.64 (0.24)		0.67 (0.17)		0.68 (0.20)	
Missing		/	0.60 (0.29)		0.64 (0.18)		0.63 (0.24)	
Random		/	0.50 (0.25)		0.52 (0.29)		0.50 (0.27)	

*Np* Non-parametric estimator, *GL* Ghosh-Lin model with no variable selection

*ρ *controls the level of correlation among covariates within the covariance matrix$\Sigma(\rho)$. RecForest was trained with fixed values for *minsplit= 5* and *nodesize=10.* 250 learning sets and one external validation set were generated for each scenario. Values closer to 1 indicate higher performance

**Table 3:** Means and standard deviations of the integrated score without a terminal event

Scenario \ Method	GL	RecForest $\:mtry=1$	RecForest $\:mtry=\sqrt{p}$	RecForest $\:mtry=\text{log}\left(p\right)$
Low dimensional$\:\left\{n=250,p=20\right\}$				
Complete,$\:\rho\:=0$	50.45 (41.00)	208.10 (102.42)	539.49 (451.96)	161.12 (87.65)
Complete,$\:\rho\:=0.2$	−29.86 (25.50)	143.23 (90.05)	449.37 (400.00)	170.45 (92.04)
Complete,$\:\rho\:=0.5$	−54.50 (33.01)	123.83 (85.78)	402.10 (375.50)	139.92 (85.37)
Missing	35.78 (17.91)	325.30 (189.63)	498.75 (415.28)	258.90 (112.14)
High dimensional$\:\left\{n=250,p=300\right\}$				
Complete,$\:\rho\:=0$	/	309.47 (134.57)	388.20 (226.95)	355.65 (117.78)
Complete,$\:\rho\:=0.2$	/	256.81 (139.33)	327.55 (160.00)	301.55 (141.18)
Complete,$\:\rho\:=0.5$	/	239.60 (125.66)	290.41 (149.59)	278.73 (134.48)
Missing	/	398.70 (229.57)	574.50 (318.75)	475.20 (213.80)

*GL* Ghosh-Lin model with no variable selection

*ρ* controls the level of correlation among covariates within the covariance matrix$\Sigma(\rho)$ . RecForest was trained with fixed values for *minsplit=5* and *nodesize=10*. 250 learning sets and one external validation set were generated for each scenario. Higher values indicate higher performance.

#### With a terminal event

 On average, 62% of the individuals experienced at least one recurrent event, 46% had at least two recurrent events, 26% had at least five recurrent events, and circa four recurrent events per individual.On average, 44% of individuals experienced a terminal event during the observation period.

Overall, performance discrepancies in terms of C-index values (Table 4) were observed when dealing with missing data or randomness, with performance being notably lower compared to complete data, as expected. In the low-dimensional setting ($\:p=20$), RecForest achieves C-index values as high as 0.82 under no correlation ($\:\rho\:=0$) with $\:mtry\:=\:log\left(p\right)$, significantly outperforming GL at 0.52. While the C-index decreases slightly with increasing correlation, RecForest remains robust, achieving values above 0.66 even at $\:\rho\:=0.5$, whereas GL fluctuates around 0.55. RecForest also outperforms GL under missing and random scenarios, maintaining C-indices in the range of 0.71–0.75 under missingness. In the high-dimensional setting ($\:p=300$), RecForest continues to perform well, with C-index values ranging from 0.69 to 0.74 across all $\:\rho\:$ levels and $\:mtry$ values, demonstrating strong discrimination even when many features are present and correlated.

Integrated scores for evaluating approaches with a terminal event are displayed in Table 5. RecForest once again shows a clear advantage, particularly in high-dimensional and correlated settings. In the low-dimensional case with a terminal event, GL’s score deteriorates significantly with increasing correlation (e.g., from 110.86 at $\:\rho\:=0$ to − 11.43 at $\:\rho\:=0.5$), indicating poor calibration under multicollinearity. RecForest, on the other hand, maintains positive and high scores across all scenarios. Even with higher $\:\rho\:$, scores remain above 250 with $\:mtry\:=\:1$, and even higher with larger $\:mtry$. In the high-dimensional case, RecForest performs strongly across the board, with scores increasing under lower correlation and remaining stable even when $\:\rho\:=0.5$. While performance under missing data shows a slight drop, RecForest still maintains robust scores, demonstrating its ability to deliver well-calibrated scores even in challenging data conditions.

**Table 4:** Means and standard deviations of the C-index with a terminal event

Scenario \ Method	Np	GL	RecForest$\:mtry=1$	RecForest$\:mtry=\sqrt{p}$	RecForest$\:mtry=\text{log}\left(p\right)$
		Low dimensional$\:\left\{n=250,p=20\right\}$			
Complete,$\:\rho\:=0$	0.52 (0.03)	0.57 (0.07)	0.79 (0.05)	0.80 (0.04)	0.82 (0.04)
Complete,$\:\rho\:=0.2$		0.57 (0.12)	0.69 (0.10)	0.76 (0.11)	0.76 (0.11)
Complete,$\:\rho\:=0.5$		0.55 (0.19)	0.61 (0.32)	0.67 (0.24)	0.66 (0.29)
Missing		0.51 (0.11)	0.73 (0.19)	0.71 (0.13)	0.75 (0.11)
Random		0.48 (0.10)	0.51 (0.19)	0.50 (0.17)	0.52 (0.15)
		High dimensional$\:\left\{n=250,p=300\right\}$			
Complete,$\:\rho\:=0$		/	0.71 (0.19)	0.69 (0.13)	0.74 (0.10)
Complete,$\:\rho\:=0.2$		/	0.71 (0.21)	0.70 (0.13)	0.74 (0.10)
Complete,$\:\rho\:=0.5$		/	0.69 (0.23)	0.70 (0.15)	0.73 (0.17)
Missing			0.64 (0.20)	0.68 (0.13)	0.71 (0.11)
Random		/	0.49 (0.22)	0.52 (0.17)	0.53 (0.20)

*Np* Non-parametric estimator, *GL* Ghosh-Lin model with no variable selection

*ρ *controls the level of correlation among covariates within the covariance matrix$\Sigma(\rho)$ . RecForest was trained with fixed values for *minsplit= 5* and *nodesize= 10*. 250 learning sets and one external validation set were generated for each scenario

**Table 5:** Means and standard deviations of the integrated score with a terminal event

Scenario \ Method	GL	RecForest$\:mtry=1$	RecForest$\:mtry=\sqrt{p}$	RecForest$\:mtry=\text{log}\left(p\right)$
Low dimensional$\:\left\{n=250,p=20\right\}$				
Complete,$\:\rho\:=0$	110.86 (75.14)	315.76 (119.41)	446.14 (410.88)	410.90 (115.54)
Complete,$\:\rho\:=0.2$	54.44 (12.80)	299.23 (140.90)	400.01 (310.15)	387.58 (306.06)
Complete,$\:\rho\:=0.5$	−11.43 (18.05)	250.39 (133.32)	385.95 (298.55)	362.05 (292.95)
Missing	112.81 (77.64)	368.62 (211.11)	406.20 (275.57)	392.85 (138.23)
High dimensional$\:\left\{n=250,p=300\right\}$				
Complete,$\:\rho\:=0$	/	547.89 (229.37)	589.14 (472.33)	628.67 (122.41)
Complete,$\:\rho\:=0.2$	/	513.80 (213.98)	582.45 (436.72)	593.15 (228.43)
Complete,$\:\rho\:=0.5$	/	458.22 (401.37)	530.57 (387.55)	601.08 (334.19)
Missing	/	392.34 (39.16)	578.52 (336.71)	512.85 (441.29)

*GL* Ghosh-Lin model with no variable selection

*ρ *controls the level of correlation among covariates within the covariance matrix$\Sigma(\rho)$ . RecForest was trained with fixed values for *minsplit=5* and *nodesize=10.* 250 learning sets and one external validation set were generated for each scenario

In summary of the simulation study, our findings illustrate RecForest superior performance across all examined scenarios. Unlike the comparator GL model, RecForest effectively addresses both missing data and high-dimensionality. Furthermore, in random scenarios, RecForest outputs randomness, implying its reliability when the input lacks discernible patterns.

## Illustrative example: the readmission data

**Fig. 3:**
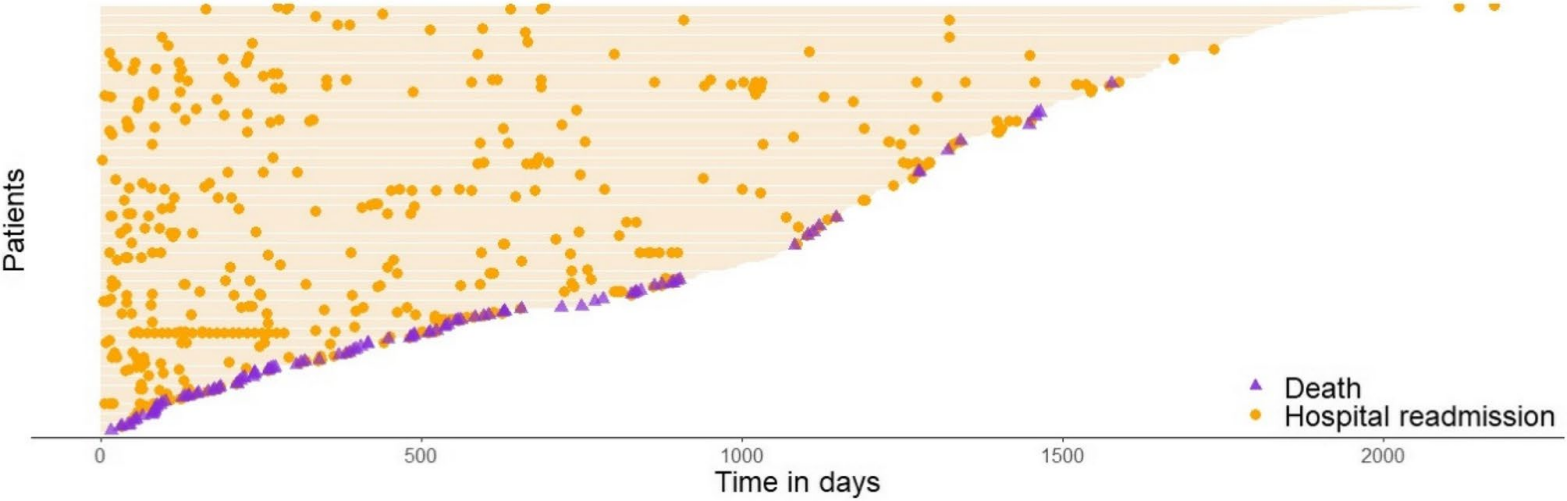
Event plot for readmission data

Readmission dataset from the frailtypack R package is widely used to demonstrate methodological principles from recurrent events analysis in presence of a terminal event [26, 41]. The data consist of multiple rehospitalizations after surgery in 403 patients diagnosed with colorectal cancer. Available factors are sex (M/F), chemotherapy treatment (Yes/No), Dukes’ tumoral stage (with levels A-B, C, and D), and Charlson comorbidity index (with levels 0, 1–2, and ≥ 3). In average, there were 1.13 (min. – max. = 0–22) hospital readmissions per patients, with 199 patients with no admission and a total of 106 deaths (Fig. 3).

In absence of an external validation set, performances were assessed with a 10-fold cross-validation procedure. We consider the following models: four multivariate Ghosh-Lin models with arbitrary combinations of factors, and RecForest. The Charlson comorbidity index was treated as a time-dependent covariate. The reference model is the non-parametric estimator.

Hyperparameter tuning was performed. explored multiple values of $\:mtry\:\in\:\{1,\:2,\:6\}$ (also derived from $\:p$ the number of covariates), along with $\:nodesize\:\in\:\{5,\:10,\:20\}$ and $\:minsplit\:\in\:\{2,\:5,\:10\}$. The best-performing model, identified through hyperparameter tuning, was obtained with $\:mtry\:=\:4$, $\:minsplit\:=\:2$, and $\:nodesize\:=\:5$. Further details are given in Appendix.

Table 6 summarizes model performance across a 10-fold cross-validation using the concordance index (C-index) and the integrated score (IScore). These metrics assess, respectively, the models’ discriminative ability and calibration relative to a non-parametric reference.

RecForest achieved the highest overall performance, with a C-index of 0.78 (SD = 0.07) and a positive IScore of 252.10 (14.69), indicating strong discrimination and improved calibration relative to the benchmark. In contrast, the best-performing parametric model (GL*), reached a C-index of 0.60 (0.06) and an IScore of 51.33 (142.63), outperforming other GL variants but still falling short of RecForest. The non-parametric estimator used as a reference model showed a C-index of 0.58 (0.05), which notably exceeded that of most GL models. This suggests that the added complexity of GL2 – GL4 did not translate into improved predictive performance and may reflect an underfitting tendency or inability to capture non-linear patterns in the data.

Comparing IScore metrics, RecForest and the non-parametric estimator are not directly comparable due to construction. The IScore values reported represent relative calibration performance with respect to a reference estimator. This makes them suitable for comparison across models, since all methods are evaluated against the same benchmark. A higher IScore thus indicates better calibration with regards to the reference model (here the non-parametric estimator). Specifically, the non-parametric reference for the integrated score in RecForest is constructed for each bootstrap sample. Integrated scores for GL models operate on the overall dataset from the ongoing fold. Consequently, IScore values from RecForest do not simply reflect the difference between error values from the non-parametric estimator and RecForest, as opposed to IScores from GL models. Besides, IScore values for RecForest indicate lower margin of errors. Among GL models, GL1 (Ghosh-Lin model with sex) exhibits a higher IScore, compared to GL2 to GL4 (GL2 = Ghosh-Lin with sex and chemotherapy; GL3 = Ghosh-Lin model with sex, chemotherapy and Dukes’ tumoral stage; GL4 = Ghosh-Lin model with sex, chemotherapy and Dukes’ tumoral stage and Charlson’s index) yield negative IScore values, indicating a worse calibration than a non-parametric estimator. RecForest reports not only positive IScore indicating better calibration than reference model, but also higher raw value. Besides, high variability is observed across all approaches.

**Fig. 4:**
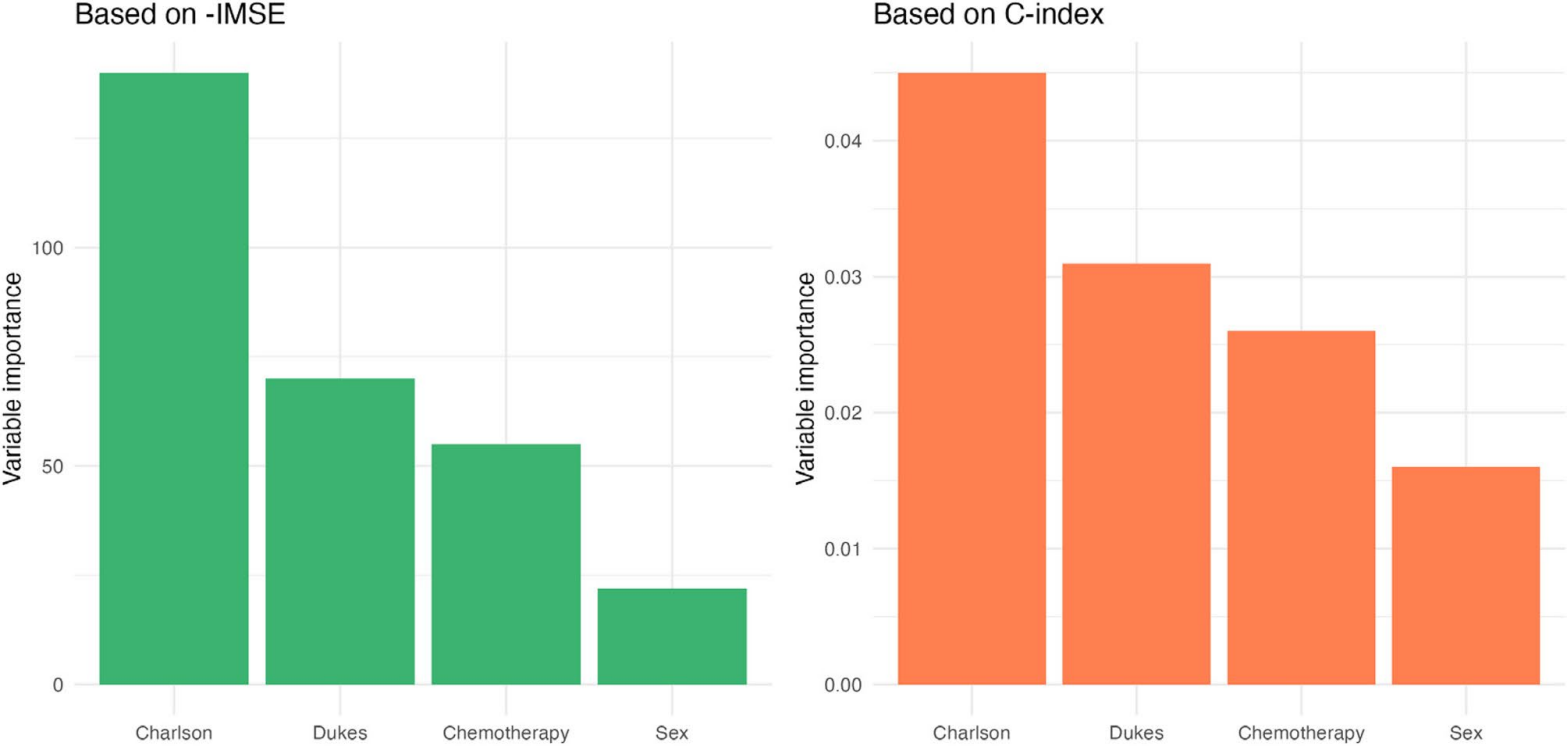
Variable importance of RecForest computed on the C-index and the opposite of the integrated MSE. Charlson refers to Charlson comorbidity index, Dukes refers to tumoral Dukes stage

Variable importance for RecForest was based on both the C-index and the opposite of the integrated MSE (Fig. 4). Most important variable identified by RecForest was the Charlson comorbidity index. Sex and chemotherapy did not seem to have an impact on the predictive performance. Variable selection enabled to reach better performance for GL* model.

**Fig. 5:**
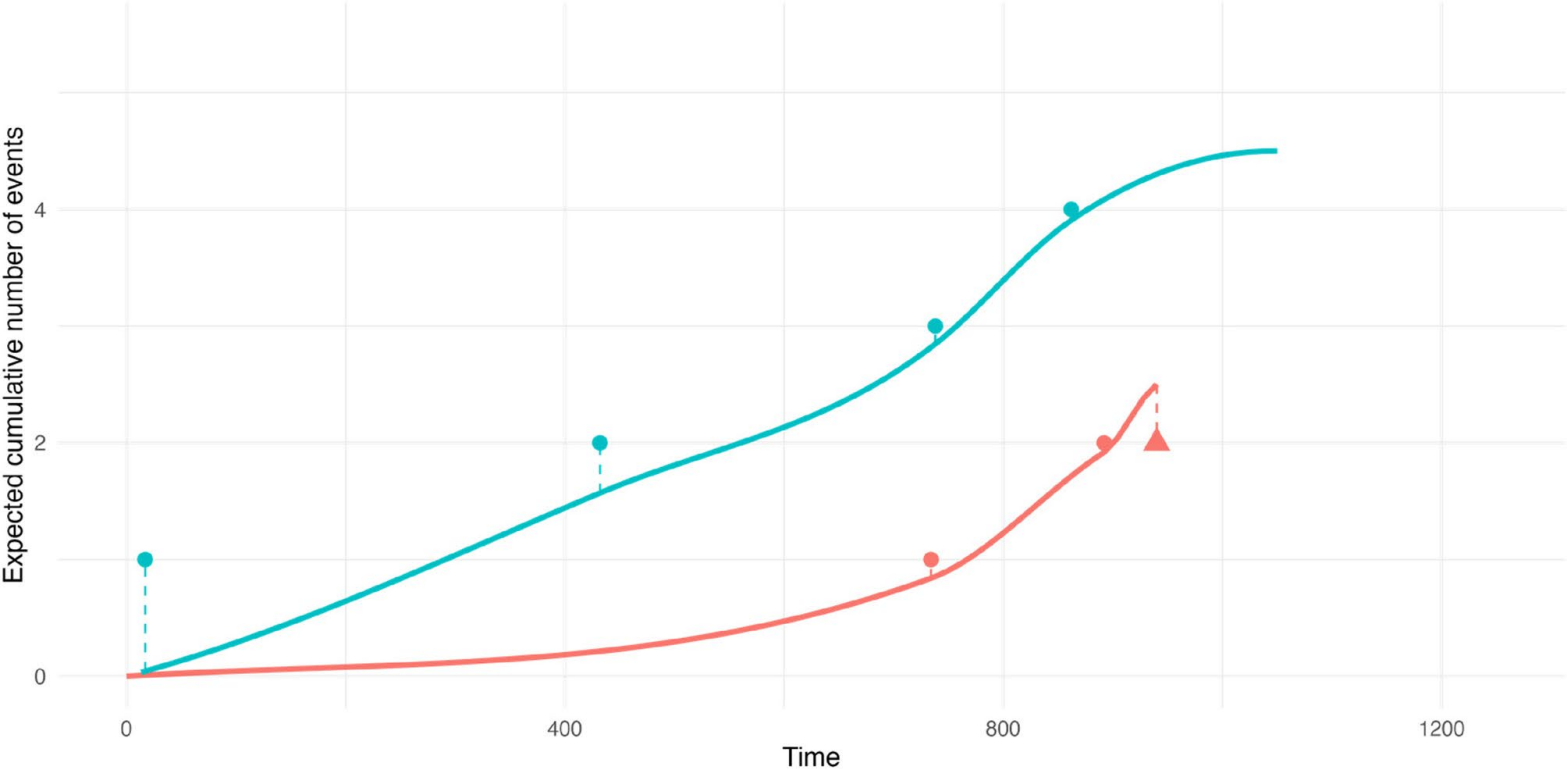
Expected cumulative number of recurrent events with RecForest for two patients, one in orange with the highest Charlson comorbidity score, and the other in blue with the lowest. Data points outside the prediction curves are observed data. Triangle indicates the patient died

Prediction curves for RecForest as the expected number of recurrent events are displayed in Fig. 5. Predictions were generated for two patients, one with the highest Charlson comorbidity score (in orange), and the other with the lowest (in blue). We observe for the patient in orange that the model overestimated the expected number of three readmissions as the patient dies after two observed readmissions. For the patient in blue, the model predictions are in line with observed events.

In terms of computational time, RecForest remains tractable for practical use. Fitting a forest with 100 trees takes approximately one minute on a standard machine (M3 Pro, 18GB RAM). In comparison, the traditional Ghosh–Lin model fits in under 2 s. While RecForest involves greater computational cost due to tree construction and recurrent event-specific splitting, this remains manageable and is offset by the model’s flexibility and ability to capture complex nonlinear effects.

**Table 6:** Means and standard deviations over the 10-fold cross-validation

Metric	Np	GL1	GL2	GL3	GL4	RecForest	GL*
C-index ↑	0.58 (0.05)	0.53 (0.08)	0.48 (0.08)	0.48 (0.07)	0.45 (0.05)	0.78 (0.07)	0.60 (0.06)
IScore ↑	ref.	39.41 (230.6)	−477.67 (348.48)	−345.62 (432.6)	−2 098.44 (541.59)	252.10 (14.69)	51.33 (142.63)

﻿Arrows indicate whether higher are lower scores lead to best performances

*Np* Non-parametric estimator, *GL1* Ghosh-Lin model with sex, *GL2* Ghosh-Lin with sex and chemotherapy, *GL3* Ghosh-Lin model with sex, chemotherapy and Dukes’ tumoral stage, *GL4* Ghosh-Lin model with sex, chemotherapy and Dukes’ tumoral stage and Charlson’s index, GL* Ghosh-Lin model with best variables from RecForest

## Discussion

We developed RecForest by extending the RSF algorithm to handle recurrent events in a survival framework, in potential presence of a terminal event, of longitudinal markers, and of missing data. To do so, the splitting rule at each node was tailormade for recurrent events analysis and mean cumulative number of events served in terminal node estimators. We characterized the performance both with discrimination and calibration by introducing a generalized C-index for recurrent event analysis and applying an innovative MSE.

In a simulation study, we compared RecForest with two baseline approaches: a non-parametric estimator and a GL model. Scenarios included variations of the presence of a terminal event, the low- or high-dimensionality of the data, multicollinearity amongst covariates, and the inclusion of missing values. In instances where missing values or high-dimensional data were present, greater variability was observed across all scenarios for both metrics. In all explored cases, RecForest demonstrated higher performances in terms of discrimination with the C-index with baseline GL models, and in terms of calibration with the IScore compared with the non-parametric estimator. The impact was quite little when introducing massive noisy data. Besides, RecForest emerged as the sole modeling approach capable of handling high-dimensionality scenarios with no prior variable selection. Furthermore, we presented a practical application using well-known open-source data, showcasing how the fine-tuning of RecForest hyperparameters leads to a more performant model. Again, RecForest exhibited superior predictive performance, achieving a C-index of 0.78 (0.07), alongside strong calibration (IScore = 252.10 (14.69)). Overall, across both simulated and real-world datasets, RecForest consistently emerged as the most effective modeling approach.

In practical applications, high-dimensional problems involving recurrent events are often sidestepped by transforming the recurrent event survival framework into alternative formats, such as an event count, a time-to-first-event endpoint, or a classification problem. However, each of these transformations may lead to the voluntary omission of valuable information. In response to this, RecForest aims to bridge a recognized gap in handling such applications, ensuring a more comprehensive and nuanced analysis of recurrent event data. Additionally, our algorithm benefits from random forests features, i.e. the ability of handling missing data or multicollinearity, and reducing overfitting thanks to bagging principle.

Additional settings can be explored to integrate a terminal event within the proposed approach. For instance, Charles-Nelson, et al. (2019) suggested working with inverse probability of survival weighting (IPSW) to compute coefficient weights in the Ghosh-Lin model, whereas we used inverse probability of censoring weighting [7]. IPSW is typically recommended when analyzing the terminal event is also of interest. Another example would be to use frailty models, either joint or additive as per Rondeau, et al. (2012) [26]. Moreover, natural extensions of random forests, serving as ensemble methods, have been widely used to improve performance through boosting techniques like Gradient Boosting, Extreme Gradient Boosting, or LightGBM [42–44]. Since all these methods are grounded in tree-based structures, they offer seamless extensions to the proposed approach, and would hence provide innovative tools for recurrent event analysis.

With regards to the MCAR mechanism adopted in our work, we acknoldge it provides a simplified benchmark to assess model robustness under incomplete data. While this assumption is strong, it allows for controlled interpretation of the missing data effects. Extending this framework to more realistic missing data mechanisms, such as Missing At Random (MAR), represents a promising direction for future work. Recent applied work in environmental science has demonstrated the practical relevance of random forest-based methods for handling complex missingness structures under MAR assumptions, including the use of multiple imputation strategies in air quality monitoring data [45].

Besides, in our work, each RecForest tree was trained on a bootstrapped sample drawn with replacement from the original dataset, with sample size approximately $\:2n/3$, following the classical random forest framework [40]. A valid alternative is subsampling without replacement, which is supported in many implementations, for instance in scikit-learn [46]. Using subsampling can be advantageous in several scenarios, such as when working with very large datasets (where sufficient tree diversity can be achieved without replacement), when maintaining a fixed class balance is important, or when addressing imbalanced datasets where more control over class representation is desired. While we did not apply subsampling in this study, we acknowledge its potential to improve computational efficiency and consider it a promising direction for future optimization of the RecForest algorithm.

Our methodology also suffers from several drawbacks. The primary limitation of random forest-like algorithms lies in the computation time, which grows with the number of trees, the dimensionality of the data and the numbers of variables selected at each tree node. Typically, our use of 250 replicated simulations with a shared external validation set, along with reported standard deviations for key performance metrics, offers insights into the variability and robustness of RecForest across scenarios. Incorporating hyperparameter tuning within each replicate could further improve performance, albeit at a significantly increased computational cost.

Second, we assumed the proportional hazard assumption of the Ghosh model, which may not universally hold in real-world settings. In our analysis, the use of a log-rank-akin test for splitting in survival trees with no terminal event presents potential limitations. As highlighted by Cui et al. (2022), traditional log-rank-based splitting rules are prone to bias when censoring is dependent on covariates, especially under non-i.i.d. conditions [47]. While in our work we make the strong assumption that the censoring process and the covariates are independent, this bias stems from the entanglement between failure and censoring distributions, leading to the selection of variables associated with censoring rather than the event process itself. The issue becomes even more pronounced in high-dimensional settings, where noise variables may carry marginal associations with the censoring distribution, thus misleading the splitting rule and compromising model consistency. Cui et al. (2023) further emphasize that such splitting bias can impair the estimation of treatment effects and highlight the importance of robust adjustments to account for censoring [48]. To mitigate this, we recommend the use of RecForest with the Ghosh–Lin splitting even without a terminal event, which involves censoring weighting. This adjustment corrects for the splitting bias by more accurately estimating within-node failure distributions, even in the absence of terminal events, and enables convergence rates to depend only on the number of true failure-related variables rather than the full covariate space.

Furthermore, variable importance measures provided do not account for potential correlations. To address this limitation, the implementation of grouped variable importance statistics is a promising avenue for further refinement [14, 49]. Nevertheless, signs of associations would still be unavailable. Another potential limitation of random forests is their static usage of features. Dynamic predictions could indeed be included as per [50, 51].

On the other hand, the issue of interpretability in machine and deep learning, particularly in digital health has garnered significant attention [52]. Several explainability methods have been proposed such as Local Interpretable Model-agnostic Explanations (LIME), SHapley Additive exPlanations (SHAP), and counterfactual explanations [53–56]. These interpretability techniques have been recently adapted for survival analysis [57–59]. Moreover, random forest-like methods offer a valuable tool for variable selection, especially in addressing high-dimensionality or obtaining hazard ratios that are intrinsically interpretable [52, 60, 61]. Approaches such as permutation-based selection, variable hunting, and iterative feature elimination serve as effective means towards this purpose [33–35].

## Software availability

Although RSF and its derivatives have mostly been implemented in both R and Python, programs for the analysis of recurrent events in a survival framework mainly rely on packages in R. Consequently, we also developed our code in R to make it available to the wider community.

The proposed method is implemented in the open-source R package recforest, available on CRAN: https://cran.r-project.org/package=recforest. This package includes functions for training RecForest models, evaluating their performance both in terms of discrimination and calibration, and generating predictions on new data. Detailed documentation and examples are provided in the package vignettes.

## Conclusion

To conclude, we introduced a new algorithm based on survival theory for recurrent events with or without a terminal event and ensemble-based methodology for learning. RecForest is readily accessible to adequately answer further clinical needs.

## Supplementary Information

Supplementary Material 1.

## Data Availability

Data are opensource under the frailtypack R package, fully available at: https://cran.r-project.org/web/packages/frailtypack/index.html. Conflicting Interests. The authors declare no conflict of interest.

## Abbreviations

C-index: Concordance index
GL: Ghosh-Lin
MSE: Mean Square Error
Np: Non-parametric
LASSO: Least Absolute Shrinkage and Selection Operator
OOB: Out-Of-Bag
RSF: Random Survival Forests
VImp: Variable Importance
