# Supplementary material for: Random survival forests for the analysis of recurrent events for right-censored data, with or without a terminal event
Source: BMC Med Res Methodol. 2025 Nov 20;25:262. doi: 10.1186/s12874-025-02678-z (PMC12636200; doi:10.1186/s12874-025-02678-z)
Supplement: Supplementary file 1 — Supplementary Material 1. [file 12874_2025_2678_MOESM1_ESM.docx]

# **Appendix**

To optimize the performance of RecForest, we conducted agrid search over key hyperparameters using the train_forest() function from the recforest R package. The goal was to identify the configuration that maximized the model's calibration, measured using the integrated score (mse_iscore). The hyperparameter search space was defined as follows:

- $mtry \in\{1, 2, 4\}$, (where *p* = 4, the number of covariates; corresponding to 1, $\surd p$, and $p$);
- $minsplit \in\{2, 5, 10\}$;
- $nodesize \in\{5, 10, 20\}$.

For each hyperparameter combination, 100 trees were grown and the Ghosh-Lin splitting rule was used to take into account both a terminal event and the Charlson comorbidity index as a time-varying covariate.

A heatmap (Figure S1) was used to visualize the variation of the integrated score across the tuning grid. Each panel represents a different $mtry$ value. Within each panel, the x-axis corresponds to $minsplit$ and the y-axis to $nodesize$. Color gradients reflect the error, where lighter colors indicate better performance (higher score and lower error).

Figure S2 displays line plots of integrated scores mse_iscore by $mtry$, with separate panels for $minsplit$ values and colored lines for each $nodesize$. This visualization further illustrates how model performance improves with increased tree complexity ($mtry=4$), particularly under low $minsplit$ and $nodesize$.

The grid search and visual analysis demonstrate that increased tree complexity and depth lead to improved model performance for recurrent event modeling in the readmission dataset. The final chosen model ($mtry = 4, minsplit = 2, nodesize = 5$) is used in subsequent analyses.


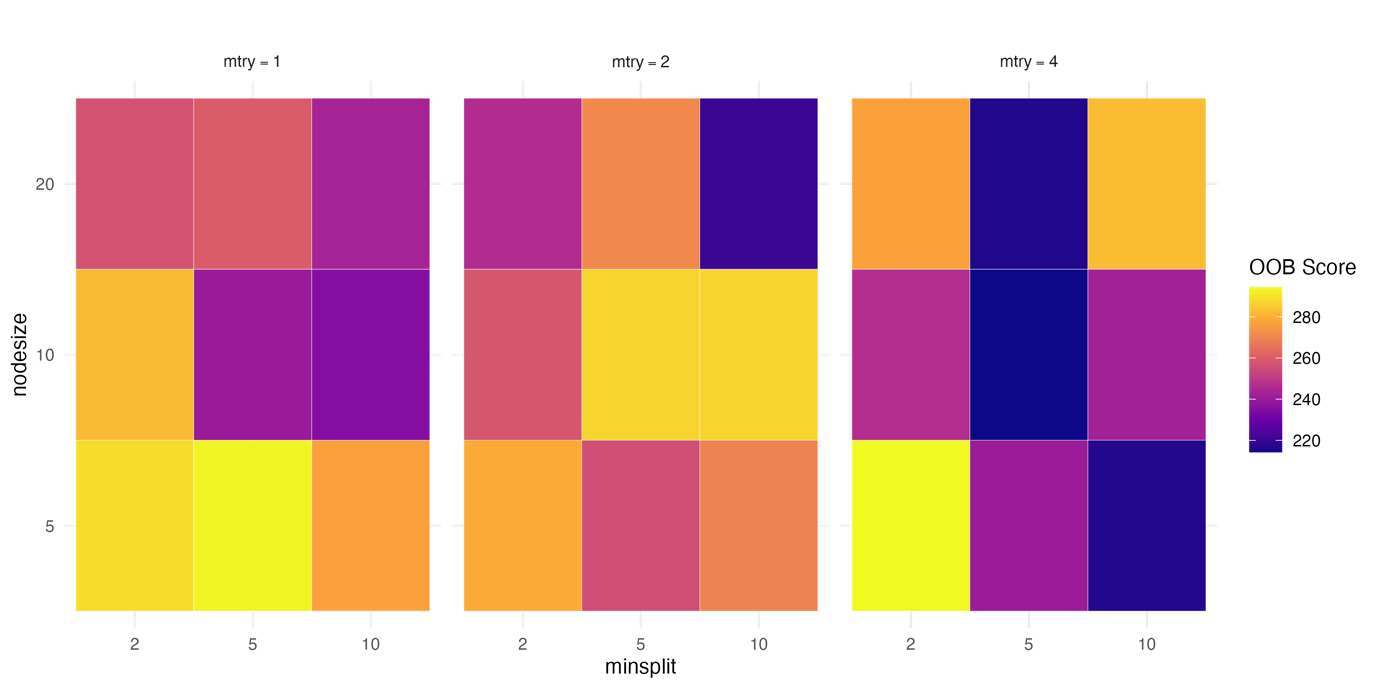


Figure S1. Heatmap of integrated scores across hyperparameter gird


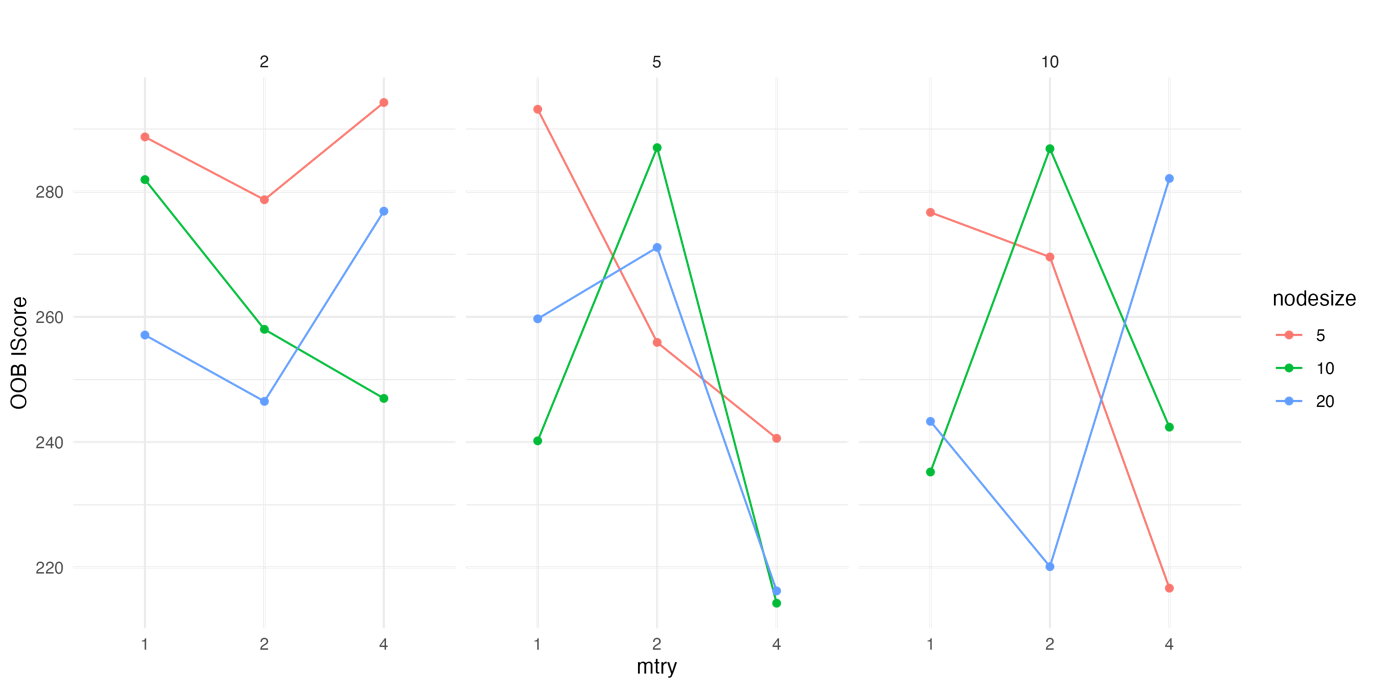


Figure S2. Line plot of integrated scores across hyperparameter grid
